# Effects of a body manipulation of Japanese martial arts on interpersonal correlation of postural sway

**DOI:** 10.1371/journal.pone.0274294

**Published:** 2022-09-12

**Authors:** Yuya Watanabe, Yutaka Sakaguchi

**Affiliations:** Department of Mechanical Engineering and Intelligent Systems, Graduate School of Informatics and Engineering, University of Electro-Communications, Chofu, Tokyo, Japan; Universiti Sains Malaysia, MALAYSIA

## Abstract

This study aimed to investigate the nature of a specific body manipulation named Suichoku-Ririku (SR) in Japanese martial arts. SR is regarded as a method to change the way of stance and to distort the balance control of the opponent, but its nature and mechanism are unknown. In the present study, we attempted to determine the effect of SR in the cases that a person stood alone (Expt. 1) and that two persons stood in contact (Expt. 2). We compared several center of pressure (COP) measures between the normal stance and SR stance conditions. When participants stood independently (Expt. 1), the COP path length, standard deviation of COP velocity and permutation entropy of the COP increased with the SR stance, which suggested that the SR maneuver destabilized a quiet stance. When two participants stood (with normal stance) in contact by wrist-holding or by a light touch (Expt. 2), their COP motions were correlated with each other, as previously reported. When one of the participants took the SR maneuver, their correlation and mutual information were maintained, denying the view that SR would diminish the interpersonal correlation of body sway. On the other hand, a fluctuation in the COP increased only for the participant taking the SR maneuver, and not for the other participant. This asymmetric effect of the SR maneuver between two participants, irrespective of maintained mutual correlation, suggest that the relationship between balance controls of two participants was partly disrupted. We discuss possible mechanisms for the present results.

## 1. Introduction

The mechanism of manipulation of the body in martial arts is unclear. The characteristics of martial arts motions have not been well described systematically or objectively. Additionally, the success or failure of martial arts actions are usually evaluated by subjective judgements of artists themselves, which causes difficulty in capturing their nature. In the present study, we focus on a specific maneuver of Japanese martial arts, called Suichoku-Ririku (SR), and investigate its nature experimentally.

Generally, martial artists gather information of the opponents by tactile or haptic information through their physical contact and wait for the appropriate opportunity to launch an attack. Therefore, haptic communication is likely to play an essential role in martial arts. This in turn means that distorting the opponent’s balance control through this communication must be effective, and the SR maneuver could be considered as one of such techniques. The SR maneuver is believed to promote independence of balance control and, as a result, to untune the opponent’s balance, though its mechanism is completely unknown (see S1 Appendix).

Motor control in human standing depends on various sensory information, including visual, somatosensory and vestibular signals [1–3]. Human postural sway is greatly reduced by touching an external object, even lightly with a fingertip (light touch effect) [4, 5]. When a person touches a moving object, postural sway is synchronized with its motion [6, 7], and the postural sways of two individuals are correlated with each other when one lightly touches the other [8–11] (interpersonal light-touch effect). These findings indicate that humans use haptic information for postural control and deal with the touched object as a reference.

The fact that the SR maneuver is regarded as a technique to disrupt dependence between two contacted persons leads to a question of whether or not SR interferes with the correlation of postural sway between two people in contact. The present study is motivated to answer this question.

Here, we explain more about the SR maneuver (see S1 Appendix for details). The term SR (meaning “vertical take-off" in English) originated from an advocate that SR involves standing as if one’s feet were lifted up vertically from the ground. SR is not a specific posture for doing some actions, but is an internal (or muscular) maneuver: It causes little body movement and can be taken with any standing posture. SR is taught with instructions such as “try to bend and extend the knees at the same time” and “try to stand on one foot for both (left and right) sides at the same time.” Naturally, people cannot actually perform these actions, but these instructions suggest that the body state of SR is realized by motor intention of simultaneous antagonistic actions of bending and extending the leg joints. In fact, antagonistic muscle pairs are activated (i.e., co-activation) around leg joints in the SR maneuver (see S2 Appendix). Nevertheless, body sensation/perception in the SR maneuver is different from that when a person simply hardens the body. Instead, SR resembles the state of the arm when a person attempts to drip one drop of water from a glass. In this situation, a person intends to perform two contradictory actions (i.e., to let water drip down and to stop the drip) simultaneously. Another example is the situation where a pianist produces a pianissimo (weak) sound. He/she attempts to strike keys (to make sound) and to stop the strike motion (not to make a loud sound). We do not mean that the SR maneuver is just the same as these daily body manipulations, but note that intending to perform antagonistic actions at the same time is never extraordinary but relatively common.

If we want to reveal the authentic nature of the SR maneuver in martial arts, we need to examine its effect in a realistic situation. However, scientifically analyzing its effect in such uncontrolled conditions is difficult. Therefore, we planned to investigate its effect in quiet standing, instead. The effect observed in quiet standing might be different from that in real martial arts actions, but this trial must be meaningful because the SR maneuver is often performed when initiating an attack from static stance.

The present study aimed to examine the effect of the SR maneuver on postural sway of an individual standing independently (Expt.1) and on correlations between postural sways of two individuals in contact (Expt. 2). To achieve this aim, we analyzed the characteristics of the center of pressure (COP). COP motion has been widely used for characterizing the nature of postural sway in quiet standing [3, 12]. Various measures on COP dynamics have been proposed to describe the nature of postural sway, and their strengths and weaknesses have been discussed [13, 14]. On the basis of these previous considerations, we adopted the following measures to compare the SR stance with the normal stance: 1) COP path length (or mean COP velocity), 2) standard deviation of COP velocity (or time derivative of COP [dCOP]), 3) permutation entropy and 4) scale exponent of detrended fluctuation analysis (DFA) for analyzing the behavior of individual participants, and 5) cross-correlation function of COP, 6) mutual information of COP and 7) detrended cross-correlation analysis (DCCA) for analyzing the relationship between two participants.

The COP path length is the length of the COP trajectory in an experimental trial, and linearly related to the mean dCOP because the mean dCOP is calculated by dividing the path length by the trial time. In quiet standing, the COP position almost agrees with the horizontal position of the center of mass (COM) of the whole body. Therefore, the path length reflects the amount of fluctuation in the COM. Similarly, the dCOP reflects horizontal motion of the COM, and its standard deviation represents fluctuation in the COM.

Various nonlinear measures have been proposed to evaluate the regularity/predictability of time series, including the correlation dimension [15], the Lyapunov exponent [16], approximate entropy [17], sampling entropy [18] and permutation entropy [19]. In this study, we adopted permutation entropy according to the suggestions by a previous study [13]. Permutation entropy showed the most consistent results among these measures in our preliminary examination. Finally, DFA is a method used for determining the statistical self-affinity of a time series [20–22]. As for the measure for the relationship between two participants, we calculated the cross-correlation, mutual information and the DCCA coefficient [23, 24]. Mutual information shows the relationship between two signals, which cannot be captured by the linear cross-correlation. DCCA is a generalization of cross-correlation based on the idea of DFA and shows the correlation in multiple time scales.

In Experiment 1, we compared these measures between the normal and SR stances when a participant stood independently. In Experiment 2, we examined these measures and relationship of COP motions when two participants stood in contact, and how they were affected when one of the participants took the SR maneuver.

## Experiment 1

### Materials and methods

#### Subjects and materials

Three participants (3 men, age range: 23–67 years) took part in this experiment. One participant was a practitioner of Japanese classical martial arts who understood SR well, and the others were the authors of this article. The authors and other laboratory members learned the SR maneuver, and some preliminary results have been published in local conferences [25, 26]. The martial artist was paid 5200 Japanese Yen for his participation in this study. This experiment was approved by the University of Electro-Communications Institutional Review Board for Human Subjects Research (#19038), and was in accordance with the ethical standards in the Declaration of Helsinki. We obtained written informed consent from all participants.

The experiment was performed in a quiet laboratory. We measured the ground reaction force using a force plate (TF-4060, Tech-Gihan, Kyoto, Japan).

#### Procedure

In each trial, participants maintained quiet standing for 40 seconds with their feet approximately 20 cm apart on the force plate. They closed their eyes and their arms hung down by their side. The experimental conditions were the normal (control) condition and the SR condition. The participants first stood in the normal stance for 40 seconds, and they then took the SR maneuver and maintained it for 40 seconds, followed by the experimenter’s signal. The participants repeated this pair of trials (i.e., block) 10 times with a short rest period between successive blocks. The whole experiment was finished in approximately 20 minutes.

Although quiet standing appears to be a simple task, it is difficult to perform it without mindedness. Most people are likely to pay unnecessary attention to specific body parts and/or do unnecessary things that they usually do not do. Some researchers imposed additional mental tasks (e.g., an arithmetic task) to control the cognitive process of the participants, although such an additional task was found to affect balance [27–29]. Therefore, we only asked the participants to repeat the task 10 times. This procedure seems effective because when continuing the quiet stance for several minutes, participants usually become accustomed to the task and are able to maintain the stance without thinking/doing unnecessary things. We discarded data from the first four blocks, and adopted data from the last six blocks for data analysis.

#### Data analysis

As described above, we only used data from six blocks for analysis. In addition, we discarded data in the first 10 seconds out of 40 second measurement in each trial. Digitally sampled ground reaction force signals (sampling frequency: 200 Hz) by the force plate were collected by a PC through a USB interface. The observed signals were filtered with a fourth-order Butterworth low-pass filter (cut-off frequency: 30 Hz) using Matlab (Mathworks, Natick, MA, USA) software. However, to calculate permutation entropy, we used the unfiltered down-sampled data (divided by 10; new sampling frequency of 20 Hz) because filtering may change non-linear signal properties [30].

The following measures were calculated: 1) the COP path length (PL), 2) the standard deviation of dCOP in the anterior–posterior (AP) and mediolateral (ML) directions (SDap and SDml), 3) permutation entropy of dCOP (PEap and PEml) and 4) alpha-exponents of DFA in the COP (DFAap and DFAml). These measures were calculated using Matlab software. When calculating permutation entropy, we set the embedding time delay = 1 and embedding dimension = 5. We performed statistical tests (Kruskal-Wallis test; non-parametric ANOVA) separately for individual participants, because we only had three participants, and we needed to examine whether common tendencies were observed among individual participants. Matlab software (“kruskalwallis” function) was utilized to perform the statistical tests. We used all data without removing the outliers.

### Results

**Fig 1:**
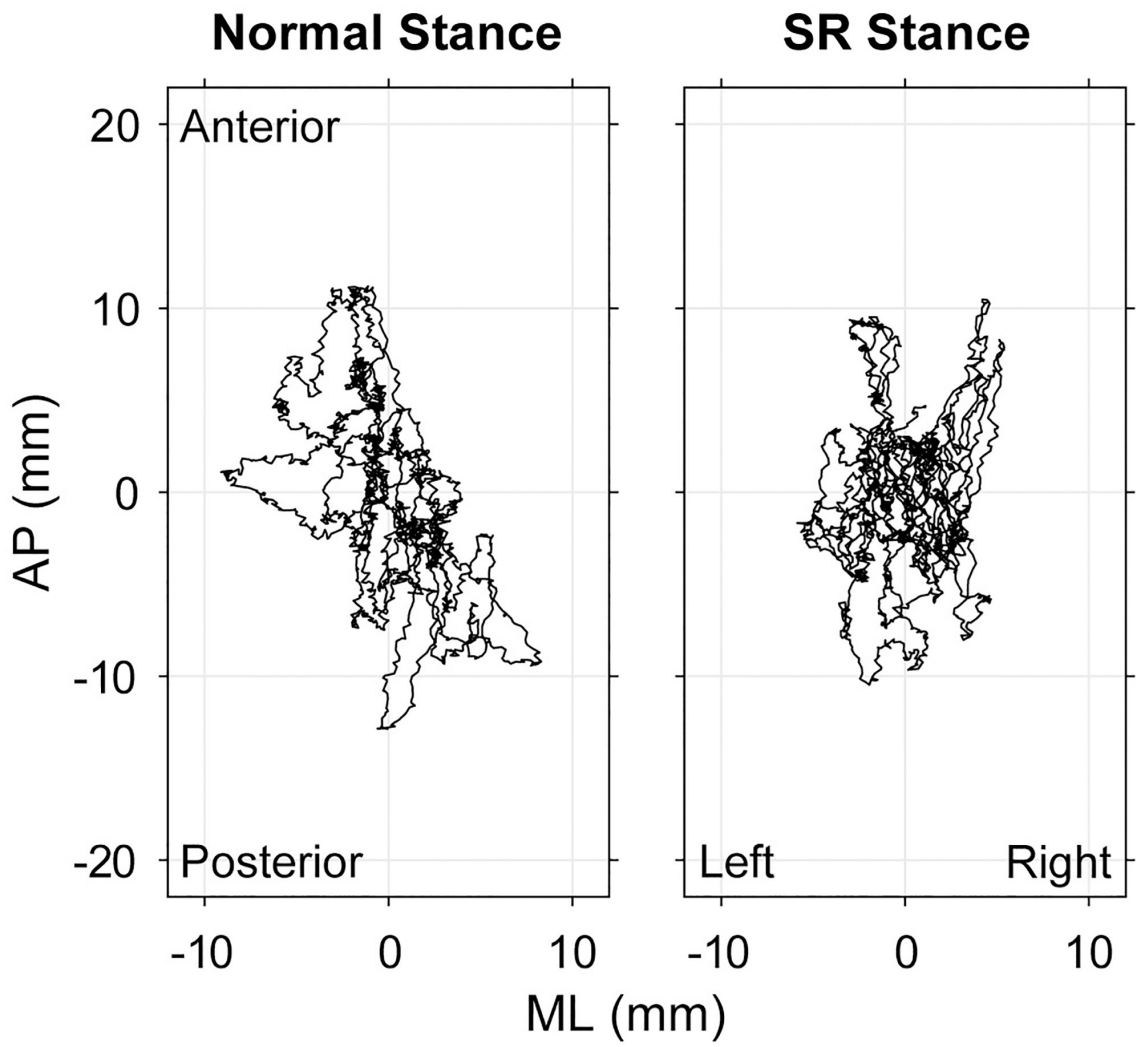
Examples of COP trajectories. Examples of the COP trajectories in the normal and SR stances are shown. No apparent difference can be found between the two results, implying that the SR stance brings no drastic change to body sway.

Examples of COP trajectories of the two conditions are shown in Fig 1. No remarkable difference was observed between them. No common tendency was found in the plots for all participants, indicating that the SR maneuver did not cause a drastic change in postural sway.

**Fig 2:**
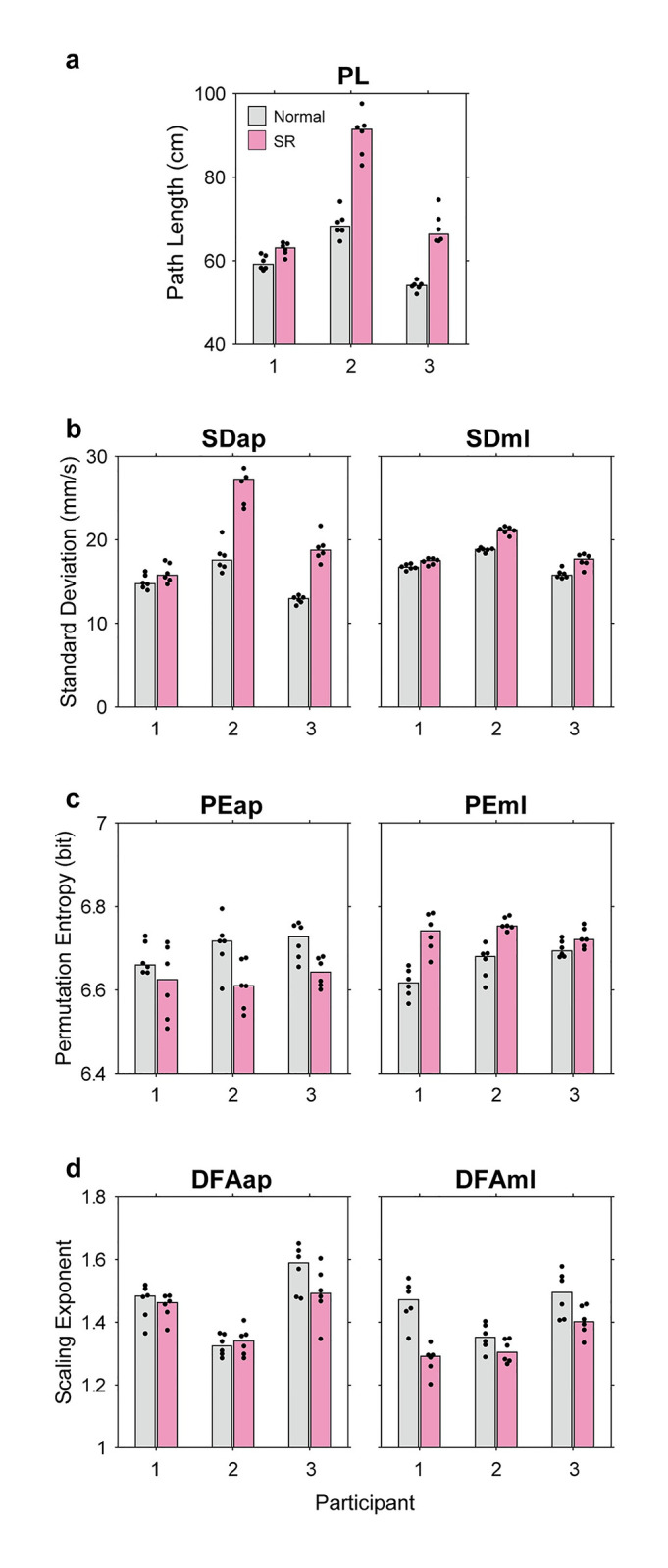
Summary of COP measures in Experiment 1. Four types of COP measures in two stance conditions are shown for individual participants: (a) COP PL, (b) standard deviation of dCOP in the AP and ML directions (SDap and SDml), (c) permutation entropy of the dCOP (PEap and PEml), and (d) alpha-exponents of DFA of COP (DFAap and DFAml). In each panel, gray and magenta bars indicate medians of experimental data in the normal and SR stances, respectively. Small dots represent data from individual trials, where their horizontal positions are fluctuated so that close dots are easily distinguished. Generally, these measures show that COP motion was more fluctuated and more irregular in the SR stance compared to the normal stance.

Fig 2 shows the four types of COP measures in the two stance conditions for the individual participants. We found the following. First, the PL was longer in the SR condition than in the normal condition in all participants (Fig 2a). This difference was significant for every participant (F[10, 1] = 6.57, p < 0.05; F[10, 1] = 8.31, p < 0.01; F[10, 1)] = 8.31, p < 0.01, Kruskal–Wallis test). Second, SDml and SDap were larger in the SR condition than in the normal condition (Fig 2b). This difference was significant for two participants for SDap (pairs of F and p values: [2.08, 0.15], [8.31, 0.0039] and [8.31, 0.0039]; Kruskal–Wallis test), and all participants for SDml (pairs of F and p values: [5.77, 0.016], [8.31, 0.0039] and [7.41, 0.0065]; Kruskal–Wallis test). These results suggested that the COP fluctuated more in the SR condition than in the normal condition.

PEap and PEml were both 6.6–6.8 bits (Fig 2c; Note that the possible maximal entropy is 6.91 bits in our setting because we set the embedding dimension to 5, the number of cases was 5! = 120, and log_2_120 = 6.91 bits). We do not further examine its absolute amount because the focus of our study was the difference between the two stances. PEap and PEml showed opposite behavior: PEap in the SR condition was smaller whilst PEml in the SR condition was greater, compared to the normal condition. There were significant differences or a tendency for significance for two participants (pairs of F and p values: [1.26, 0.2623], [8.31, 0.0039] and [5.03, 0.025] for PEap, and [8.31, 0.039], [5.03, 0.025] and [3.10, 0.0782 for PEml; Kruskal-Wallis test).

**Fig 3:**
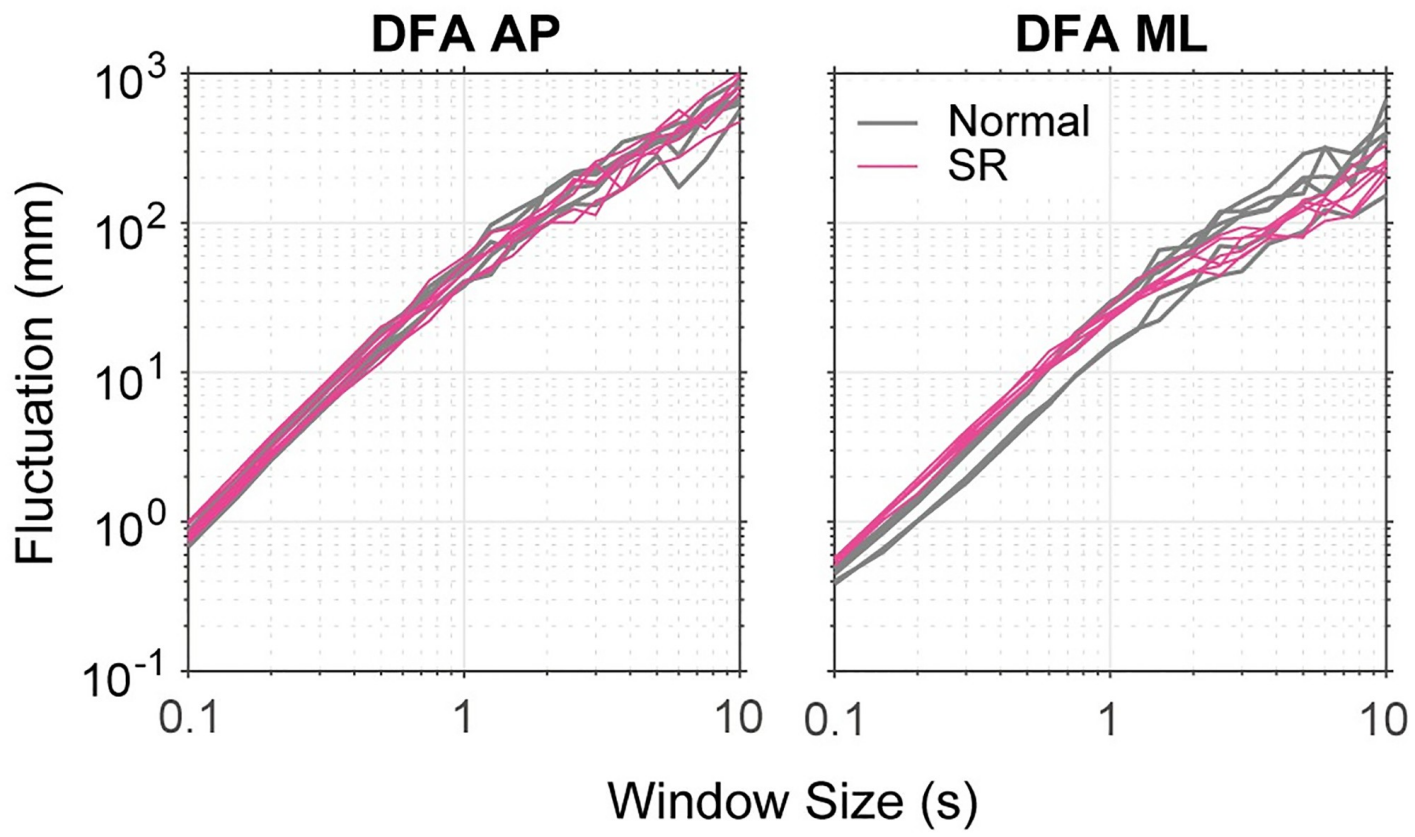
Example of DFA results. Relationship between the window size (time-scale) and fluctuation is depicted for one participant. Left and right panels shows the results in AP and ML directions, respectively. In each panel, the gray curves show the results of individual trials in the normal condition while the magenta curves show those in the SR condition. These curves are almost linear, but in the right panel (i.e., ML direction), the magenta curves bend around the window size of 1 second whilst no such bent can be found for the gray curves, implying that SR stance brought some change in postural sway.

Finally, DFAap and DFAml ranged from 1.2 to 1.6 (Fig 2d; Note that exponent close to 1.5 means that the signal is a Brownian noise [20]). DFAml was smaller in the SR condition than in the normal condition, but no tendency was found in DFAap. These differences were significant or showed a tendency only for DFAml (pairs of F and p values: [8.31, 0.039], [3.10, 0.0782] and [3.10, 0.0782]), but not for DFAap ([0.64, 0.4233], [0.026, 0.8728] and [2.08, 0.1495]). Fig 3 shows the relationship between the window size (time scale) and fluctuation in Participant 1. These plots are almost linear, but in the ML direction, the broken curves (i.e., the SR condition) bend and their slopes decline at approximately a window size of 1 second. Since the scaling exponents were estimated from the slope of these curves, smaller values of DFAml in the SR condition were due to this bend. This difference in slopes suggested that the SR stance led to a certain change in postural sway.

### Discussion

This experiment examined the COP’s characteristics of the SR stance compared with those of the normal stance. Although no drastic change was found in the COP trajectory (Fig 1), most COP measures indicated that the SR stance led to more fluctuation in COP behavior (Fig 2). The SR stance resulted in a significantly increased PL, SDap, SDml and PEml in all participants. Additionally, the SR stance decreased DFAml in two of three participants.

Greater fluctuation induced by the SR stance appears somewhat strange considering that antagonistic muscle pairs of the leg joints are co-activated in the SR stance (see S2 Appendix). Generally, co-activation of antagonistic muscles increases the viscoelastic property (i.e., stiffness and damping) of muscles and joints [31–33], which presumably leads to a decrease in postural sway. A possible reason for this result is that co-activation may change the mode (or structure) of balance control. In human balance control, generally, the AP direction is more important than the ML direction [3]: Balance in the ML direction is easily stabilized by two legs especially when two feet are separated as in the present experiment. There are two major strategies of balance control in the AP direction, namely the ankle strategy and the hip strategy [3]. In the ankle strategy, the muscles around the ankle joints act to control the body as an inverted pendulum. In the hip strategy, the hip moves the COM to the AP direction. It can be conjectured that if the ankle and hip joints are less mobilized by co-contraction [31], people might control their balance using other joints or other degrees of freedom and that this exceptional control structure may alter COM motion, resulting in more fluctuation in COP behavior.

This view may explain the asymmetric results observed in permutation entropy and DFA. The SR maneuver increased PEml, but deceased PEap. Since permutation entropy reflects the regularity of the signals [19], this result means that COP motion became more regular in the AP direction but less regular in the ML direction. The reduction in PEap seems consistent with the view that the leg joints are less mobilized the AP direction, and that maybe a new control structure is formed, bringing more irregular COP motion in the ML direction.

With regard to the scaling exponent of DFA, the effect of SR was only observed in DFAml, and not in DFAap. Moreover, DFA curves in the ML direction bent around the window size of 1 second in the SR condition. This result seems consistent with our conjecture that the SR maneuver changes control structure which alters the COP motion in the ML direction. Our view is only speculation at the present and we have no concrete support for this idea. However, at least, the present finding clearly indicates that the SR maneuver altered the nature of quiet stance.

## Experiment 2

The second experiment was performed to examine the effect of the SR maneuver on the interpersonal correlation of COP motions when two persons are contacted either by close contact or by a light touch. Previous studies have shown that the COP motions are mutually correlated when two persons are contacted even by a light touch [8–11]. This means that that haptic information alone (i.e., even without physical force) can modulate the controls of quiet stance of two persons. In this experiment, we asked whether the effect of SR maneuver equally appeared in the close-contact and light-touch conditions. The result may give us a clue to explore how the SR maneuver of one person affects the balance control of the other (i.e., physical force vs. haptic communication).

### Materials and methods

#### Subjects and materials

Ten participants (10 men, age range: 21–67 years) took part in this experiment. Three of them were the same as those in Experiment 1, and the remaining participants were graduate or undergraduate students at the University of Electro-Communications. The experiment was approved by the University of Electro-Communications Institutional Review Board for Human Subjects Research (#19038), and was in accordance with the ethical standards in the Declaration of Helsinki. We obtained written informed consent from all participants. The martial artist was paid 5200 Japanese Yen, and student participants were paid 1200 Japanese Yen for their participation in the study.

The experimental material was the same as that in Experiment 1, except that we used two same-type force plates: One participant stood on one while the other stood on the other.

#### Procedure

The experiments were conducted in 15 pairs formed from 10 participants. One of the pairs (active participant; A-participant) was chosen from three participants who could perform SR maneuver and the other (passive participant; P-participant) was chosen from the remaining participants.

An experimental block consisted of five trials, each of which corresponded to an experimental condition. The task required the participants to stand quietly for 40 seconds with their eyes closed, as in Experiment 1. In the first trial, both participants stood independently in the normal stance with their arms hanging down and facing each other (independent / normal stance (I-N) condition). In the second trial, both participants were in the normal stance, but flexed their elbows so that the forearms were almost horizontal, and the A-participant softly grabbed the wrists of the P-participant by both hands (close contact / normal stance (C-N) condition). In the next trial, the A-participant performed the SR maneuver while holding the wrists of the P-participant (close contact / SR stance (C-SR) condition). In the fourth trial, both participants were in the normal stance, and the A-participant lifted their right forearm and lightly touched the side of the pelvis of the P-participant by the index finger (light touch / normal stance (LT-N) condition). In the last trial, the A-participant performed the SR maneuver while lightly touching the P-participant (light touch / SR stance (LT-SR) condition). The order of the conditions in one block was fixed for all participant pairs. The participants repeated the tasks for 10 blocks. They took a short rest between succeeding blocks as required. The whole experiment took approximately 50–60 minutes to complete.

#### Data analysis

As in Experiment 1, we used data from the last six blocks for further analysis, while data in the last 30 seconds (out of 40 second measurement) were used. We adopted the following measures for analyzing the individual data: 1) PL, 2) SDap and SDml, 3) PEap and PEml, and 4) DFAap and DFAml. With regard to the interpersonal relationship between the COP motions, we used cross-correlation (CCap and CCml), mutual information (MIap and MIml) and DCCA coefficients (DCCAap and DCCAml) in the AP and ML directions. These measures were calculated using Matlab software. As in Experiment 1, filtered data (low-pass filter with a cut-off frequency of 30 Hz) were used for calculating these measures, except that unfiltered down-sampled data (20 Hz) were used for calculating permutation entropy. In the calculation of MIap and MIml, we quantized COP values into 40 discrete intervals and calculated the probabilities for every interval.

We performed the Kruskal–Wallis test for testing the effect of experimental conditions using the median of six blocks as a representative value of each participant pair. We used all data from 15 participant pairs without removing the outliers.

### Results

**Fig 4:**
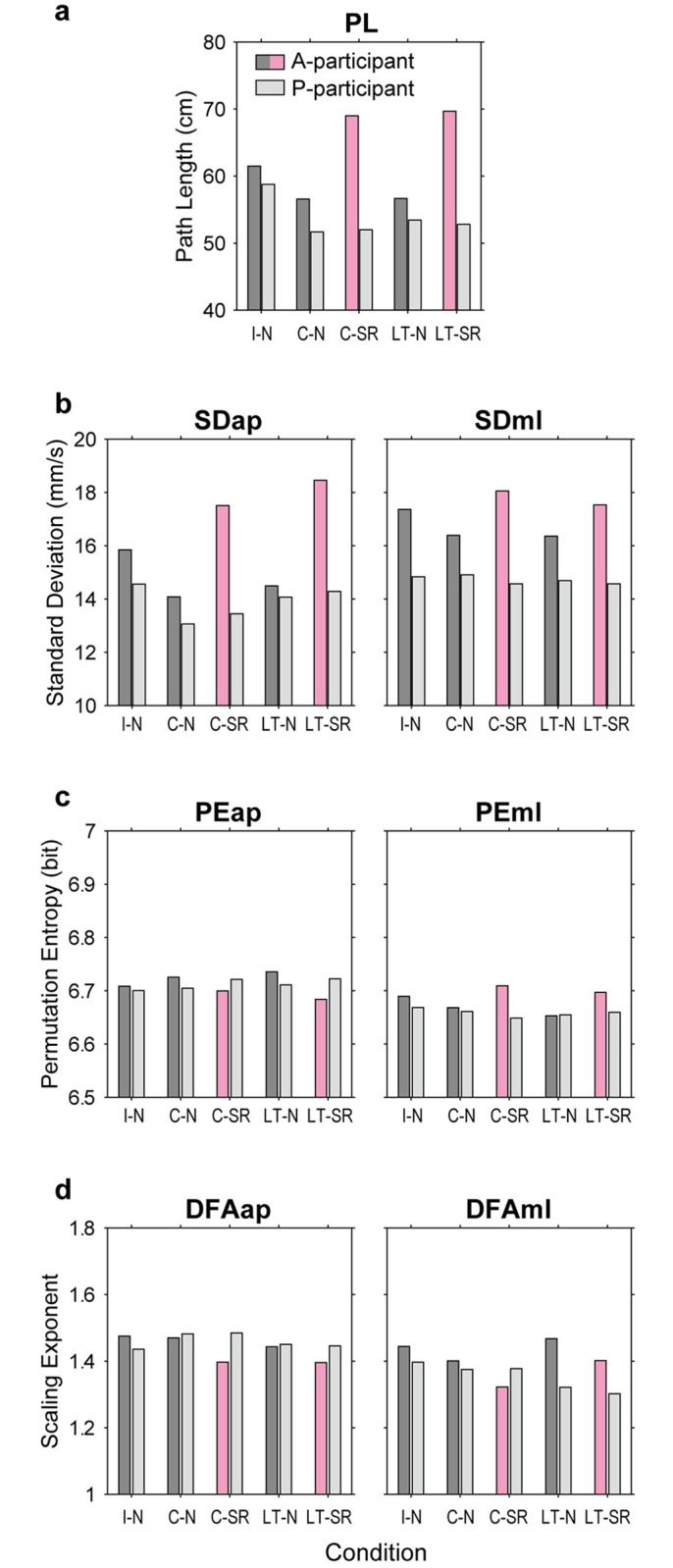
Summary of COP measures in Experiment 2. Four types of COP measures in five experimental conditions are shown for A-and P-participants: (a) COP PL, (b) standard deviation of the dCOP in AP and ML directions (SDap and SDml), (c) permutation entropy of the dCOP (PEap and PEml), and (d) alpha-exponents of DFA (DFAap and DFAml). In each panel, the horizontal and vertical axes show the experimental conditions and measure values, respectively. The dark gray bars and light gray bars represent the inter-participant median values of A-participants and of P-participants, respectively, while the magenta bars indicate the conditions that A-participants took the SR maneuver. The representative value of each participant pair was given by the median of six blocks.

Fig 4 shows four COP measures of A- and P-participants in five experimental conditions. We found that the PL was shorter in the C-N and LT-N conditions than in the I-N condition in A- and P-participants. The same relationship was also found for SDap and SDml, although the difference in SDml was small. These results suggested that either close contact or a light touch diminished body sway in both participants. Next, the PL of A-participants was longer in the C-SR and LT-SR conditions than in the C-N and LT-N conditions, but this difference was not observed for P-participants. A similar tendency was also found for SDap and SDml. These findings indicates that the SR stance increased fluctuation of COP motion in A-participants, but not in P-participants. We found that the effect of the experimental conditions was significant for PL (F[70, 4] = 26.82, p < 0.001), SDap (F[70, 4] = 33.92, p < 0.001) and SDml (F[70, 4] = 11.37, p < 0.05) in A-participants, but not significant for PL (F[70, 4] = 4.32, p = 0.3649), SDap (F[70, 4] = 7.8, p = 0.0992) or SDml (F[70, 4] = 0.57, p = 0.9664) in P-participants (Kruskal–Wallis test). Post-hoc multiple comparison tests showed significant differences in the PL, SDap and SDml for C-N vs. C-SR, C-N vs. LT-SR, LT-N vs. LT-SR and LT-N vs. C-SR (all p < 0.05) in A-participants.

**Fig 5:**
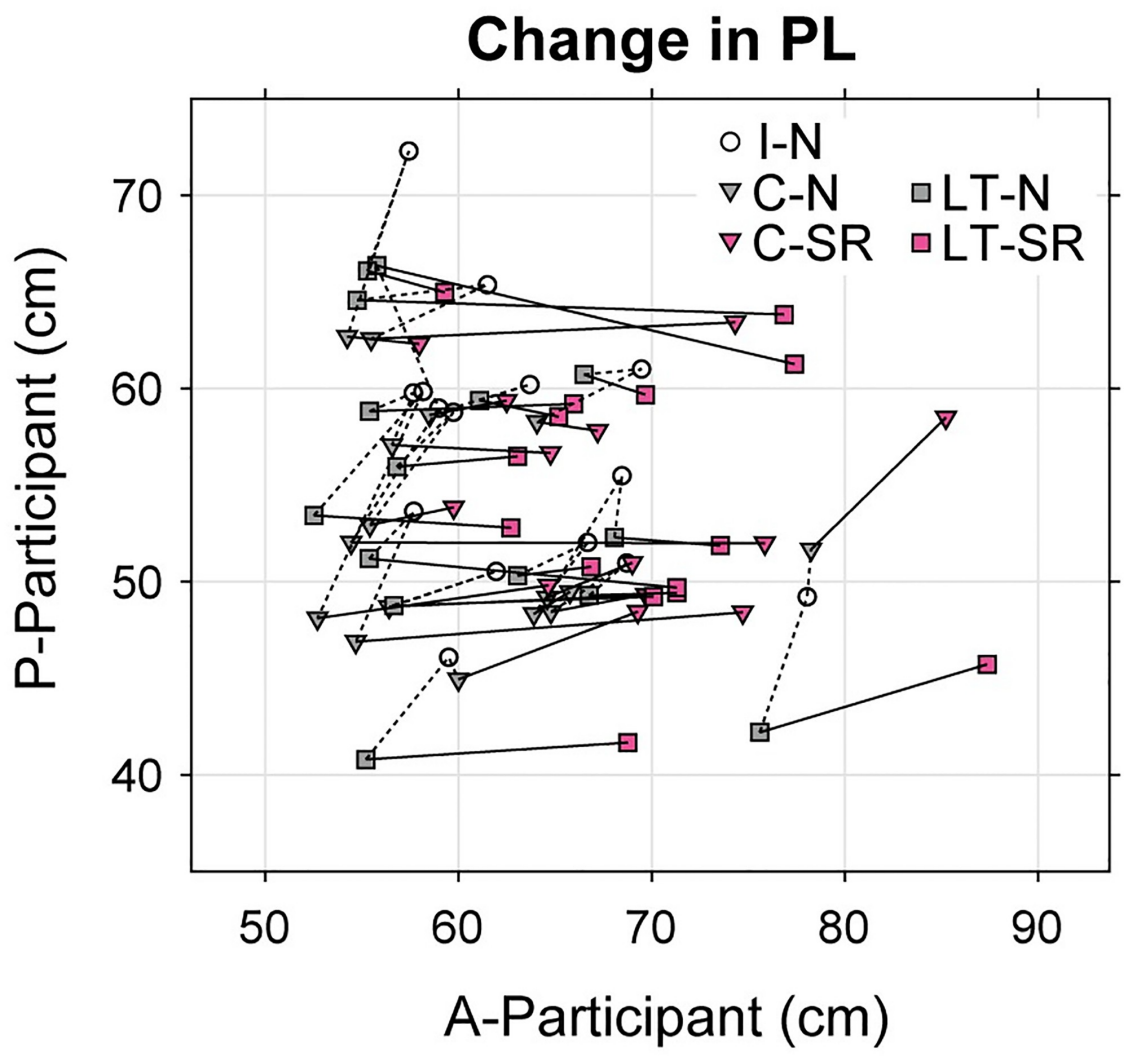
Relationship between changes in PL for paired participants. This figure summarizes the changes in the PL for every participant pair where the horizontal and vertical axes show the PLs of A-participants and of P-participants, respectively. The open circles represent the I-N condition, the gray and magenta triangles represent the C-N and C-SR conditions, and the gray and magenta squares represent the LT-N and LT-SR conditions, respectively. Each line segment represents a result from one participant pair: Solid line segments connect the normal and SR conditions (C-N vs. C-SR and LT-N vs. LT-SR) while broken segments connect the I-N, C-N, and LT-N conditions. Note that most solid ling segments (connecting the normal and SR conditions) are aligned horizontally, meaning that the SR maneuver did not increase the PL for P-participants but increased for the A-participants for almost all pairs.

Fig 5 shows the relationship between changes in the PL in two participants. The solid line segments, connecting the normal and SR conditions (C-N vs. C-SR and LT-N vs. LT-SR), are aligned in the horizontal direction, which clearly shows that the SR maneuver increased the PL in A-participants, but not in P-participants, for almost all pairs.

PEap and PEml ranged from 6.6 to 6.8 bits (Fig 4c), similar to Experiment 1. An apparent effect of the experimental conditions was found only in A-participants, and this effect appeared in the opposite directions between PEap and PEml. For A-participants, PEap was smaller and PEml was greater with the SR maneuver (C-SR and LT-SR) than with a normal stance (C-N and LT-N). This asymmetric effect of the SR maneuver in the AP and ML directions is the same as that in Experiment 1. In contrast, no consistent changes were found in PEap or PEml in P-participants. The effect of experimental conditions was significant for PEap (F[70, 4] = 21.83, p < 0.001) and PEml (F[70, 4] = 14.6, p < 0.01) in A-participants, but not for PEap (F[70, 4] = 3.16, p = 0.5316) or PEml (F[70, 4] = 1.85, p = 0.7632) in P-participants (Kruskal–Wallis test). Post-hoc multiple comparison tests showed significant differences in PEap for I-N vs. LT-SR, C-N vs. LT-SR and LT-N vs. LT-SR (all p < 0.05), but a significant difference in PEml was only found for LT-N vs. C-SR (p < 0.05) in A-participants.

With regard to the scaling exponent of DFA (Fig 4d), the effect of the condition was more evident in DFAml than in DFAap, as in Experiment 1. In A-participants, generally, the exponent was smaller in the contact conditions than in the individual condition. Additionally, the exponent was even smaller in the SR conditions (C-SR and LT-SR) than in normal conditions (C-N and LT-N). However, in P-participants, no consistent tendency was found, except that the exponent was smaller in the light touch conditions (LT-N and LT-SR) than in the close contact conditions (C-N and C-SR). The effect of the experimental conditions was significant for DFAap (F[70, 4] = 16.38, p < 0.05) and DFAml (F[70, 4] = 30.9, p < 0.001) in A-participants, but not significant for DFAap (F[70, 4] = 5.16, p = 0.2713) or DFAml (F[70, 4] = 7.9, p = .0954) in P-participants (Kruskal–Wallis test). Post-hoc multiple comparison tests showed significant differences in DFAap for I-N vs. LT-SR and C-N vs. LT-SR (both p < 0.05) and in DFAml for I-N vs. C-SR, LT-N vs. C-SR and LT-SR vs. C-SR (all p < 0.05) in A-participants.

**Fig 6:**
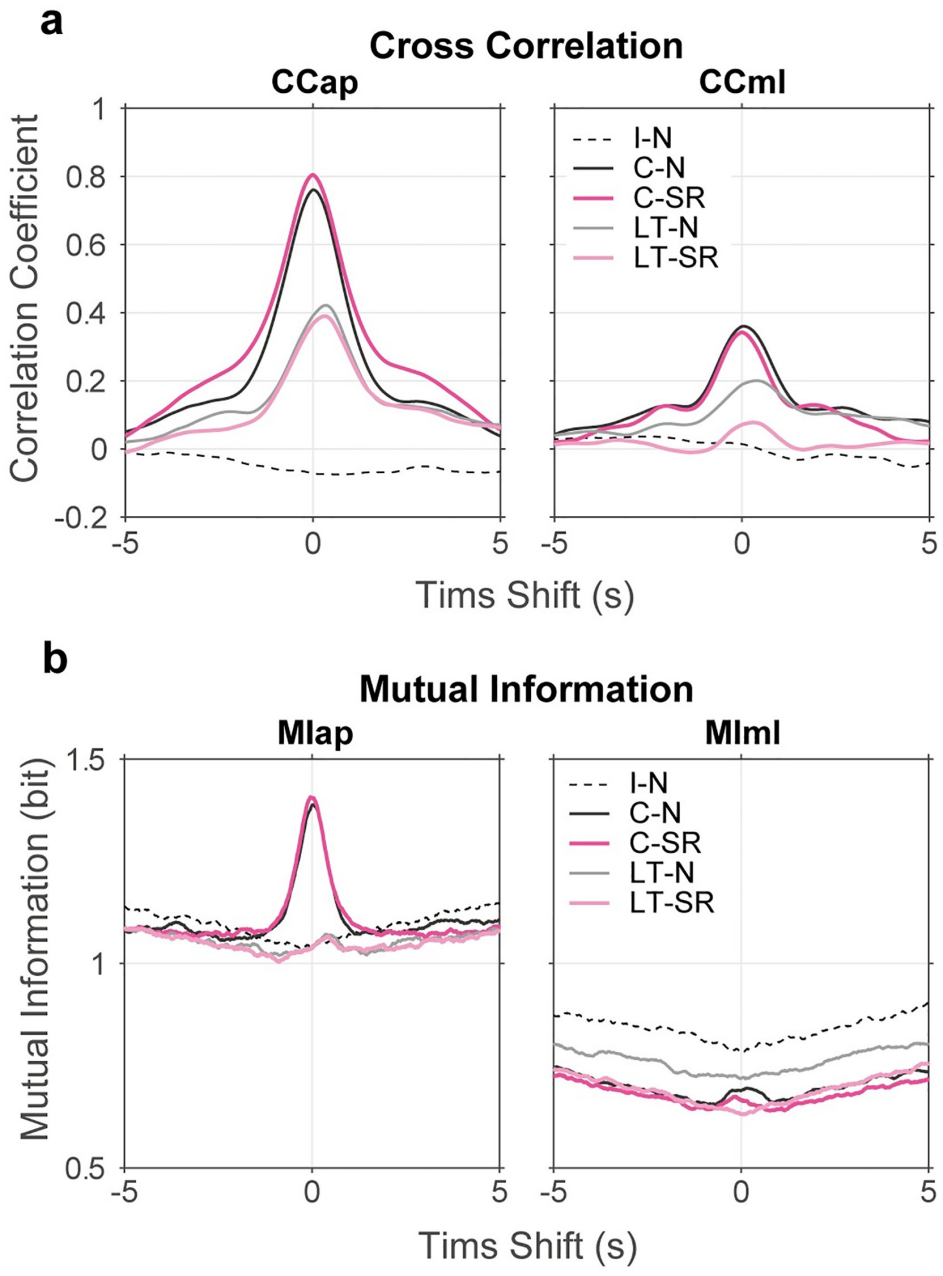
Relationship between COPs of two participants. (a) Averaged CCap and CCml functions and (b) averaged MIap and MIml functions between COPs of two participants are shown for five experimental conditions. Broken curves represent the results of the I-N condition. The dark gray and magenta curves represent the results of the C-N and C-SR conditions, and the light gray and magenta curves represent the results of the LT-N and LT-SR conditions, respectively. These curves were obtained by averaging the functions over pooled data of 15 participant pairs × 6 blocks. The horizontal axis represents the time shift between the signals from A- and P-participants where a positive value means that the signal from P-participant is in advance of that from A-Participant. All CCap, CCml, MIap and MIml show positive peaks in two contact conditions. Peak values of these functions were the larger in the close contact conditions (C-N and C-SR) than in the light touch conditions (LT-N and LT-SR). The peak time shift was almost zero in the close contact conditions but was around 300 ms in the light tough conditions. There was little difference between the normal stance and the SR stance in CCap, but in CCml, remarkable difference was found between two stances in the light-touch condition.

Fig 6 shows the averaged CCap, CCml, MIap and MIml functions in five conditions where the average was calculated from pooled data (i.e., 15 pairs × 6 blocks). As expected, the plots of CCap and CCml are approximately at the zero level in the I-N condition. In the same manner, the amounts of MIap and MIml are also maintained at approximately the same value in the I-N condition. In contrast, CCap and CCml showed clear positive peaks in the other conditions, which indicates that the COP of two participants was positively correlated in both AP and ML directions. Moreover, the peak values of CCap and CCml were larger in the close contact conditions (C-N and C-SR) than in the light touch conditions (LT-N and LT-SR). This finding indicates that close contact resulted in a stronger relationship between the participants than a light touch. We also found that the peak values of CCap were greater than those of CCml, and that MIap showed only a slight increase whilst MIml showed little change. These findings suggested that the relationship between COP motions was formed primarily in the AP direction. The peak time shift was almost zero in the close contact conditions, but was approximately 300 ms in the light touch conditions. This means that the body sway of two participants was synchronized in the close contact conditions whilst in the light touch conditions, the sway of the A-participant lagged behind that of the P-participant by 300 ms. This difference was observed in all CCap, CCml, MIap and MIml. Furthermore, there were little differences in CCap, MIap and MIml between the normal and SR stance conditions (C-N vs. C-SR and LT-N vs. LT-SR). However, we should note that CCml appeared to be smaller in the LT-SR condition than in the LT-N condition. Notably, the finding that MIml had the largest value in the I-N condition was unexpected, but it might have been due to some spurious effect caused by hidden characteristics of COP signals.

**Fig 7:**
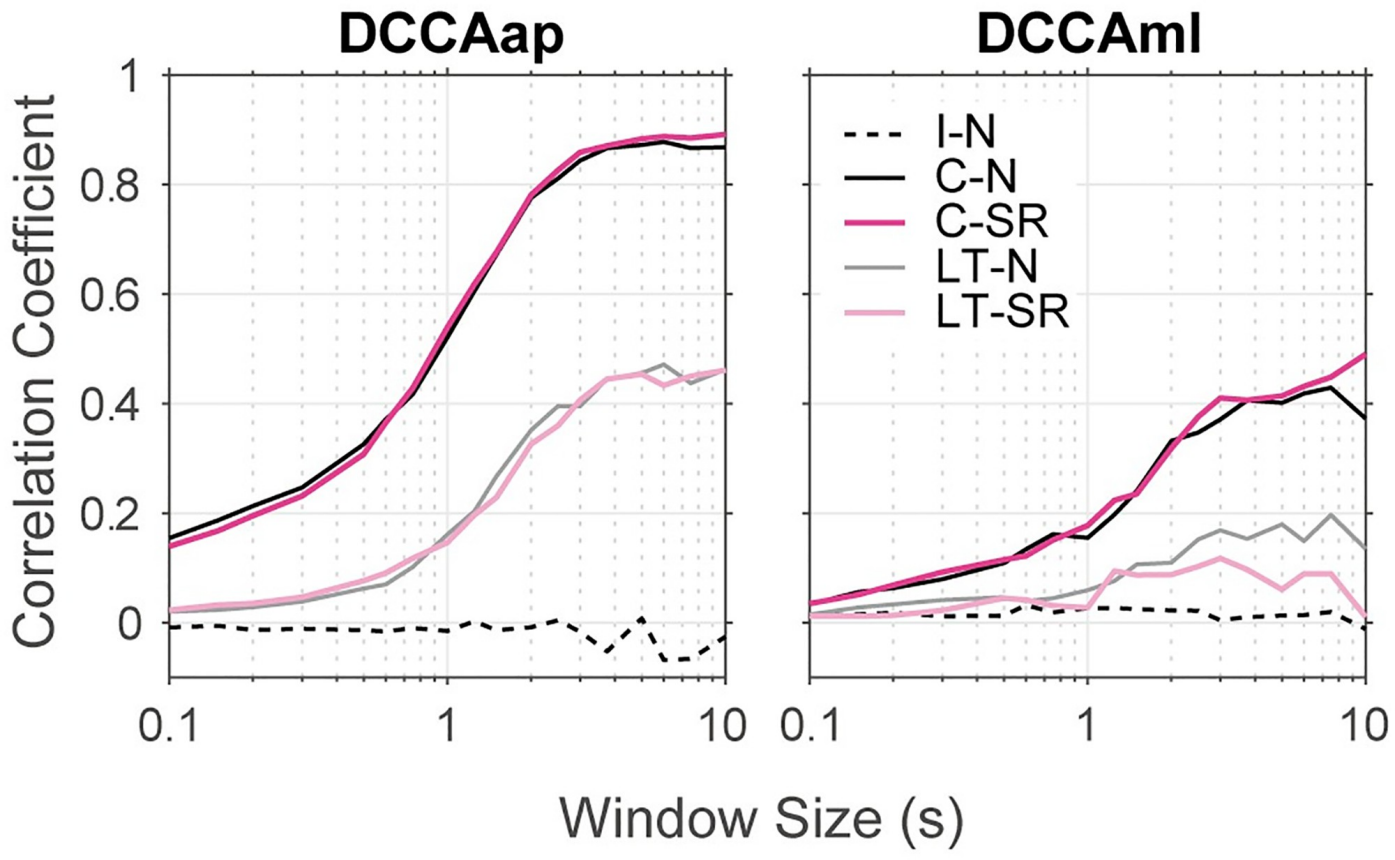
Result of DCCA. Averaged DCCAap and DCCAml coefficients are shown. Broken curves represent the results of the I-N condition. The dark gray and magenta curves represent the results of the C-N and C-SR conditions while the light gray and magenta curves represent those of the LT-N and LT-SR conditions, respectively. Average was calculated over pooled data of 15 pairs × 6 blocks. As we found in Fig 6, correlation between two participants were maintained by the SR maneuver except DCCAml in the light-touch conditions.

Finally, Fig 7 shows the result of DCCA, which was consistent with the nature of the cross-correlation function shown in Fig 6. DCCAap and DCCAml coefficients were approximately zero for all time ranges in the I-N condition, but it increased with greater window sizes in the other conditions. The correlation was greater in the close contact conditions (C-N and C-SR) than in the light touch conditions (LT-N and LT-SR). This greater correlation for a longer time scale is consistent with a previous report, which showed that strong coupling was observed under a frequency of 0.4 Hz in the light touch effect [6]. There was little difference between normal stance and the SR stance conditions (C-N vs. C-SR and LT-N vs. LT-SR), except that a considerable reduction in DCCAml was found in the time range over 3–4 seconds (i.e., 0.3 Hz) in the LT-SR condition compared with that in the LT-N condition.

### Discussion

Experiment 2 examined the effect of the SR maneuver on the relationship between COP motions of two contacted people. First, we showed that the PL, SDap, and SDml were decreased when two participants were in contact either by wrist-holding or by a light touch (Fig 4). This suggests that the body sway is stabilized by interpersonal touch, which is consistent with previous studies [7, 9]. Notably, this stabilization was observed for both A-participants and P-participants: Stabilization occurred when A-participants lightly touched P-participants, as in the situation when two participants symmetrically touch each other [8, 9]. We also showed that, in the light touch condition, the correlation peak was located at a time shift of approximately 300 ms (Fig 6). This finding appears to be consistent with a previous finding that the temporal change in the COP was delayed with a change in touch force by approximately 300 ms [5].

Second, this experiment showed that when two participants were in contact, the SR maneuver increased the fluctuation in COP in A-participants, but not in P-participants. The SR stance significantly increased the PL, SDap, STml, DFAap and DFAml in A-participants, but caused little changes in P-participants. This asymmetric effect between A-participants and P-participants was clearly shown in the change in PL depicted in Fig 5. An increase in COP fluctuation in A-participants is consistent with the result when participants stood independently (Expt. 1). However, surprisingly, this increase was not propagated to P-participants. This asymmetric result seems peculiar considering that either close contact or a light touch stabilized the COP fluctuations in both A- and P-participants. The reason why P-participants were insensitive to greater postural sway in A-participants only in the SR condition is unknown.

Third, the results of cross-correlation, mutual information and DCCA coefficients showed that COP motion was correlated in contact conditions (Figs 6 and 7). Especially, CCap, CCml, MIap, and MIml were all greater in the close contact conditions (C-N and C-SR) than in the light touch conditions (LT-N and LT-SR). On the other hand, the SR maneuver had little effect on these measures. This result apparently denies the view that the SR maneuver weakens the interpersonal correlation of postural sway. However, one exception is that the SR maneuver diminished CCml in the light touch condition (not in the close contact condition). This change was also observed in the result of DCCAml as a reduction of coefficients in the range around 0.3 Hz. This suggests a possibility that the effect of the SR maneuver of the A-participant may be transferred to the P-participant through haptic communication channel because it is thought that a light-touch effect is mediated by haptic communication [7, 9].

Finally, we discuss the asymmetric phenomena between the AP and ML directions. Specifically, the effect of the SR maneuver appeared in opposite directions for PEap and PEml (Fig 4c). In the light touch condition, the SR maneuver reduced CCml but not CCap (Fig 6a). Additionally, a clear peak existed in MIap, but not in MIml for either contact condition (Fig 6b). Interpreting these results is difficult. The reason why interpersonal touch gave greater effects on CCap and MIap (than CCml and MIml) is presumably because the AP direction is more crucial in human postural control than the ML direction [3]. It can be speculated that this can explain why the linkage of postural control brought by inter-personal contact (either close contact or a light touch) is more evident in this direction. On the other hand, the result that the effect of the SR maneuver was observed in the ML direction is perhaps related to the result of Experiment 1 that the SR maneuver decreased the DFA exponent only in the ML direction. In the discussion of Experiment 1, we have pointed out the possibility that the leg joints were less mobilized by the muscle co-contraction, and this may change the structure of balance control. If we accept this view, though there is no objective ground, the results of two experiments suggest that the SR maneuver may alter the roles of AP and ML directions in balance control of the A-participant, and this change may be conveyed to the P-participant through haptic communication. Anyhow, we have no decisive idea for explaining this asymmetric phenomenon. To elucidate its mechanism, we have to know more about the mechanism of interaction between balance controls of two contacted persons.

## General discussion

### Summary

In the present study, we investigated the effects of a specific body maneuver used in Japanese martial arts, called SR, on the postural sway of quiet standing and on the relationship of postural sway of two people in contact. In Experiment 1, we showed that the SR maneuver increased postural sway when participants stood independently. Changes in the PL, SDap, SDml and PEml indicated that COP motion fluctuated more in the SR stance than in the normal stance. In Experiment 2, we examined the effect of the SR maneuver when two participants touched by wrist-holding or a light touch. The COP motions of two participants were correlated by the contact, but cross-correlation and mutual information were minimally affected by the SR maneuver, especially in the AP direction. The effect of the SR maneuver was only observed in the ML direction when two participants were contacted by a light touch. Therefore, we conclude that the SR maneuver did not reduce the interpersonal correlation of COP motions. However, curiously, the SR maneuver increased fluctuation in the COP only in A-participants and had little effect on P-participants, which suggested that the SR maneuver partly reduced the linkage between postural controls of two persons. It is difficult to figure out the reason for these contradictory observations (i.e., maintained correlation and dissociated fluctuations), but we try to discuss some more on this issue in the next section.

### Possible mechanism

In the Introduction section, we mentioned that in the martial arts community, SR is believed to degrade the dependence of balance control between two contacted people. Although meaning of this statement is not clear, it suggests a possibility that SR has an effect to decouple the balance controls of two people. However, it is unlikely that the decoupling is caused by a breakdown of the physical or haptic communication channel because the result of Experiment 2 clearly showed that cross correlation and mutual information were maintained with the SR maneuver. Therefore, we should postulate that the SR maneuver does not alter the communication channel itself, but that it changes the balance control of A-participants, which modulates the balance control of P-participants through the communication channel.

In the Discussion of Experiment 1, we have proposed a hypothetic view that the SR maneuver creates a specific body state with which a person controls the balance in an unusual manner. Specifically, the SR maneuver may inhibit ankle and hip strategies by the co-contraction of antagonistic muscle pairs around the leg joints, and alter the structure of balance control, which could be observed as greater COM fluctuations and asymmetric properties between the AP and ML directions. If we accept this view, it can be imagined that the change in COM fluctuations of the A-participant may be conveyed to the P-participants through physical/haptic communication, but may not alter the structure of balance control of the P-participants. In other words, A- and P-participants are interacted with each other while their balance controls have different strategies, and as a result, the effect could differently appear between A- and P-participants. This is only speculation at the present; future studies should analyze the postural sway of the whole body, as well as simulating with a control system model, in order to examine its validity.

### Limitations

To the best of our knowledge, this is the first report on an experimental examination of the SR maneuver. However, there are a number of limitations and unanswered questions.

First, we only examined three participants who performed the SR maneuver in the present study. Although there may be concern about the generalization of the present findings, the same limitation is often encountered in studies dealing with uncommon motor skills. Importantly, 15 pairs of participants showed similar tendencies in Experiment 2 (Fig 5). This consistent result indicated that the present findings are reliable and reproducible. We expect that accumulating empirical results on this topic will resolve this limitation.

Second, the situation in the present experiment is different from the actual martial arts situation. One difference is that two martial artists do not maintain quiet standing for 40 seconds. Additionally, martial artists never maintain the SR maneuver for such a long time: They occasionally use it to disturb the opponent’s balance. In the present study, we adopted quiet standing to obtain reliable measures and to examine the interpersonal relationship. Because of this methodological issue, we cannot deny the possibility that our study may have failed to capture some essential effects of the SR maneuver that are prominent in actual martial arts situations. This problem might be resolved by analyzing the transient phenomenon when A-participants change from a normal stance to the SR stance. At present, we cannot perform such an investigation owing to the difficulty in controlling experiments, but this limitation may be overcome by future experimental innovations.

Finally, we understand only a little of the biological mechanism of the SR maneuver. The martial arts maneuver needs to be examined from various viewpoints to understand its mechanism and functioning. This difficulty is common to other research on sensorimotor skills whose body manipulation cannot be designated in an objective manner. The integration of various methodologies, from an empirical examination to computational modeling, is required to move forward with these challenging studies.

## Supporting information

S1 Appendix Additional information on SR maneuver. We give some detailed information on the SR maneuver, including its history, effect and application.
(DOCX)

S2 Appendix Muscle activity caused by SR maneuver. We show the surface EMG signal from six leg muscles to demonstrate the muscular activities in the SR maneuver. The muscle activities of most muscles increased by the SR maneuver (compared to the normal stance), suggesting that antagonistic muscle activities are increased in the SR stance. However, the relative magnitude among different muscle pairs differed between the participants.
(DOCX)
