# Supplementary material for: Effects of a body manipulation of Japanese martial arts on interpersonal correlation of postural sway
Source: PLoS One. 2022 Sep 12;17(9):e0274294. doi: 10.1371/journal.pone.0274294 (PMC9467308; doi:10.1371/journal.pone.0274294)
Supplement: S1 Appendix — We give some detailed information on the SR maneuver, including its history, effect and application. (DOCX) [file pone.0274294.s001.docx]

# S1 Appendix

Here, we explain some details of the SR maneuver. This maneuver was developed and named “Suichoku-Ririku” by Yoshinori Kono, an independent researcher/practitioner of classical Japanese martial arts, in 2003. He has not belonged to any specific school of martial arts, but explored and studied various techniques by investigating other artists’ behavior, as well as inventing/discovering new techniques by himself (He has published many books and DVDs on his techniques (e.g., [1])). The SR maneuver is one of such techniques. Some descriptions on SR can be found in his blog [2, 3].

According to personal communication with Akio Nakajima, a disciple of Kono, Kono studied feet manipulation around 2002-2003, inspired by a method of feet manipulation in walking (called “flat-lift flat-fall”) of a master of Chinese martial arts (Baguazhang). He explored body manipulation for moving the feet with the soles kept horizontal, which led to a prototype of the SR maneuver. In this sense, the SR maneuver may be regarded as a variation of “flat-lift flat-fall” technique of Chinese martial arts; one of its essence is to homogenize the ground reaction force over the sole by the flat contact to the ground. However, Kono further explored this maneuver and found it effective not only in walking but also in other actions. Actually, simultaneous antagonistic muscle activation (i.e., co-contraction) in the SR maneuver has an effect of reducing the play of the hip and leg joints and stabilizing the trunk. As an application of this nature, he demonstrated that this maneuver was effective when one tried to assist a disabled\aged person (i.e., care receiver) to stand up from the floor: With the SR maneuver, one could lift up the care receiver more stably and effortlessly. This technique was generalized by Shin-Ichiro Okada, a physical therapist, as a method of body manipulation in care giving [4]. In addition, it was found that SR modulated the opponent’s balance control when two persons were in contact. It is empirically known among martial artists that balance controls of two contacted persons are mutually interacted and, resultantly, one’s body manipulation induces reflective (i.e., involuntary) body motions of the other. Therefore, it is expected that if one takes the SR maneuver and changes the way of quiet stance, it would cause some reflective motions of the other. However, this view is a kind of “folk science” and has never been examined by rigorous scientific methodologies.

In sum, although the SR maneuver originated from keeping the feet flat in walking, it is no longer in forming a specific feet posture. Rather, its essence seems in adjusting antagonistic muscle forces of the legs so as to realize a uniform distribution of the ground reaction force over the soles together with removing the play of hip/leg joints. This manipulation causes collateral effects over the whole body (i.e., stabilization of the trunk and change in balance control), which can be effectively utilized in martial arts techniques as well as daily motor tasks.

1. Kono Y. Body manipulation learned from classical martial arts. Iwanami Shoten; 2014 (in Japanese).

2. Kono Y. “Zuikan-Roku” (“daily thoughts” in English). 2003 Jun 24 [Cited 2022 July 30]. Available from https://www.shouseikan.com/zuikan0306.htm (In Japanese).

3. Kono Y. “Zuikan-Roku” (“daily thoughts” in English). 2003 Jul 1 [Cited 2022 July 30]. Available from https:// www.shouseikan.com/zuikan0307.htm (In Japanese).

4. Okada S. An introduction to care giving with classical martial arts techniques. Igaku Shoin; 2006 (In Japanese).
