# Supplementary material for: Effects of a body manipulation of Japanese martial arts on interpersonal correlation of postural sway
Source: PLoS One. 2022 Sep 12;17(9):e0274294. doi: 10.1371/journal.pone.0274294 (PMC9467308; doi:10.1371/journal.pone.0274294)
Supplement: S2 Appendix — We show the surface EMG signal from six leg muscles to demonstrate the muscular activities in the SR maneuver. The muscle activities of most muscles increased by the SR maneuver (compared to the normal stance), suggesting that antagonistic muscle activities are increased in the SR stance. However, the relative magnitude among different muscle pairs differed between the participants. (DOCX) [file pone.0274294.s002.docx]

# S2 Appendix

We measured the surface EMG signal from six leg muscles to demonstrate the muscular activities in the SR maneuver. The target muscles were the gastrocnemius (GA), tibialis anterior (TA) (extensor and flexor of ankle joint), rectus femoris (RF), semitendinosus (S) (extensor and flexor of knee joint), interior oblique (IO) and quadratus lumborum (QL) (extensor and flexor of hip joint). Surface EMG signals were detected with active electrodes, which were placed on the belly of the muscles. Another electrode was attached onto the top of the left knee for a reference. Before attaching the electrodes, the skin was cleaned with ethanol and scrubbed with a paste (SkinPure; Nihon-Koden, Tokyo, Japan) for reducing the contact resistance. Detected signals were amplified and sampled by a bio-amplifier (Polymate AP1132; Teac, Tokyo, Japan; sampling frequency: 2 kHz). The signals were filtered with a band-pass filter (band range from16 to 200 Hz) built in this amplifier.

Three participants, who performed the SR maneuver in the main experiment, participated in the EMG measurement. They maintained quiet standing for 40 seconds in the normal and SR conditions, just the same as in Experiment 1. The participants repeated the task 10 times, and we adopted data from the last six trials for analysis. Since this task was stationary, we simply calculated the temporal average of rectified surface EMG signals between10 and 40 seconds of an experimental trial. We did not measure maximal voluntary contraction because we were primarily interested in the relative difference between two stance conditions, rather than the absolute magnitude of muscle activities. We did not calculate co-contraction index.

The figure below summarizes the relative muscle activities of the SR stance (i.e., activity in the normal stance is 1) in three participants. The activities of most muscles increased by the SR maneuver, although their relative magnitude among different muscles differed between the participants. In Participant 1, co-activation of antagonistic muscle pairs was observed in the knee and hip joints in the SR stance, while the activity of muscles around the ankle joint was similar to that in the normal stance. In Participant 2, in contrast, co-activation was observed around the ankle joint, but not around the remaining joints. In Participant 3, co-activation was found around the knee and ankle joints, but not around the hip joint. Therefore, muscle activities varied among the different participants. The fact that the consistent effects of the SR maneuver observed in the main experiments irrespective of these incompatible muscle activations between three participants suggests that co-contraction over the leg joints, rather than activities of specific muscles, may be essential in the SR maneuver. Here, we should note the possibility that unmeasured muscles are also activated by the SR maneuver. Considering that the SR maneuver causes no body movement, joint torque generated by flexorsmust be offset by that generated by some extensors. Therefore, even when an imbalanced surface EMG signal was observed between an antagonistic muscle pair (e.g., GA vs. TA in Participant 2), it is reasonable to postulate that other muscles are simultaneously activated to balance the joint torque. Overall, we consider that antagonistic muscle activities are increased in the SR stance. Quantitative examination of the muscle activities of the SR maneuver is open for future investigation.


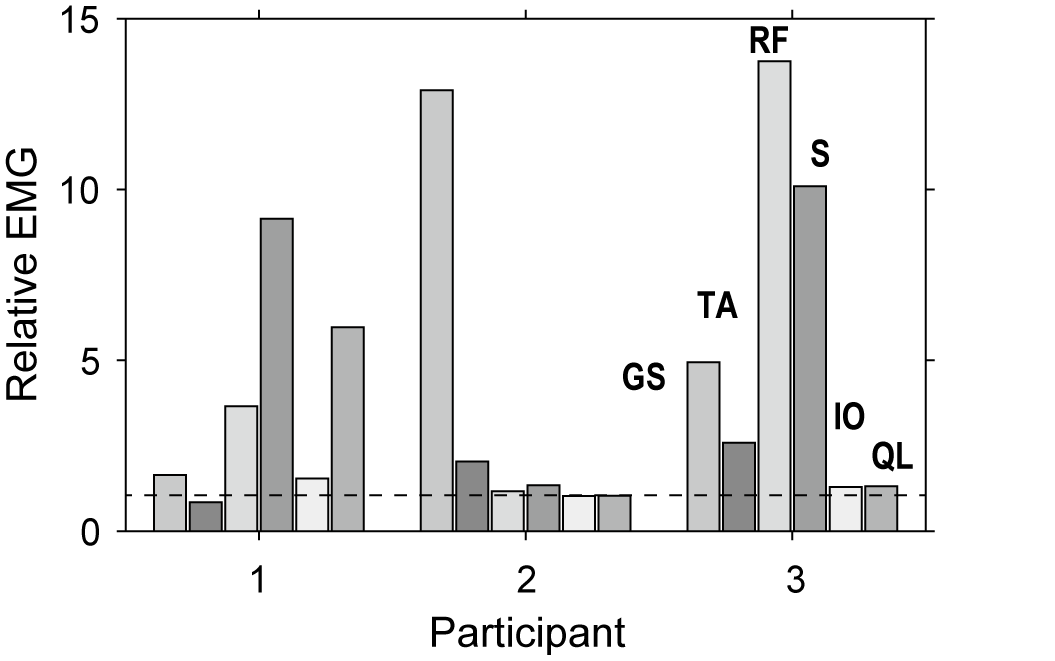


**Figure: Muscle activity in normal and SR stances.** Relative muscle activities of six muscles around the leg joints in the SR stance are depicted separately three participants. Each bar height represents the ratio of averaged sEMG level in the SR stance to that in the normal stance. Target muscles were gastrocnemius (GA), tibialis anterior (TA) (extensor and flexor of ankle joint), rectus femoris (RF), semitendinosus (S) (extensor and flexor of knee joint), interior oblique (IO) and quadratus lumborum (QL) (extensor and flexor of hip joint) of the right leg. The broken line indicates level of 1, meaning that the muscle activity in the SR stance was the same as that in the normal stance. Although the muscle activity pattern over different muscles are considerably different between three participants, it can be commonly seen that some of antagonistic muscle pairs are co-activated in the SR stance.
